# A Novel Approach for Evaluating the Influence of Texture Intensities on the First Magnetization Curve and Hysteresis Loss in Fe–Si Alloys

**DOI:** 10.3390/ma17163969

**Published:** 2024-08-09

**Authors:** Daniele Carosi, Alessandro Morri, Lorella Ceschini, Alessandro Ferraiuolo

**Affiliations:** 1 Department of Industrial Engineering—DIN, Metallurgy Group, Alma Mater Studiorum—Università di Bologna, 40126 Bologna, Italy; alessandro.morri4@unibo.it (A.M.); lorella.ceschini@unibo.it (L.C.); 2 Marcegaglia Ravenna s.p.a., Via Baiona, 141, 48123 Ravenna, Italy; alessandro.ferraiuolo@marcegaglia.com

**Keywords:** hysteresis, mathematical modeling, recrystallization, texture, electrical steels

## Abstract

This paper examines the relationship between the magnetization behavior and crystal lattice orientations of Fe–Si alloys intended for magnetic applications. A novel approach is introduced to assess anisotropy of the magnetic losses and first magnetization curves. This method links the magnetocrystalline anisotropy energy of single crystal structures to the textures of polycrystalline materials through a vectorial space description of the crystal unit cell, incorporating vectors for external applied field and saturation magnetization. This study provides a preliminary understanding of how texture influences magnetic loss rates and the first magnetization curves. Experimental results from Electron Back-Scattered Diffraction (EBSD) and Single-Sheet Tests (SSTs), combined with energy considerations and mathematical modeling, reveal the following key findings: (i) a higher density of cubic texture components, whether aligned or rotated relative to the rolling direction, decreases magnetic anisotropy, suggesting that optimizing cubic texture can enhance material performance; (ii) at high magnetic fields, there is no straightforward correlation between energy losses and polarization; and (iii) magnetization rates significantly impact magnetization loss rates, highlighting the importance of considering these rates in optimizing Fe–Si sheet manufacturing processes. These findings offer valuable insights for improving the manufacturing and performance of Fe–Si sheets, emphasizing the need for further exploration of texture effects on magnetic behavior.

## 1. Introduction

Magnetic materials are extensively used in commercial applications, including transformers, electrical motors, solenoids, and generators. Among the soft magnetic materials, Non-Oriented Grain (NGO) Fe–Si alloys are the most widely used due to their cost-effectiveness and availability [1,2,3,4,5,6,7]. Recently, the demand for higher magnetic performance in automotive electric motors has driven research towards reducing the magnetic energy loss in these steels when subjected to an external magnetic field.

It Is well known that microstructural features such as mean grain size, defects, crystallographic texture, and precipitates influence the magnetic domain movement and, consequently, the magnetic behavior of the material [1,2,3,4,5,6,7]. Researchers are thus intensively studying these effects, particularly how to improve the microstructure of Fe–Si alloys during production.

Several studies have highlighted that in Fe–Si steels, crystallographic textures significantly impact the permeability of the first magnetization curve and the area of the hysteresis loop. Specifically, a higher number of crystals with <111> directions aligned with the external applied field results in lower magnetization at a given field intensity and reduced maximum permeability. Conversely, the <100> directions have the opposite effect [1,3,5,7,8].

Research on hysteresis loss has considered various aspects of material textures, examining losses at specific values of external fields or magnetic polarization. Key parameters include the following: a generic energy texture parameter, which correlates linearly with loss [9]; average magnetocrystalline energy [10]; texture parameter $A_{\theta }$, dependent on the intensities of relevant magnetic texture components [11]; texture parameter $A_{\alpha }$, related to the rolling direction and magnetization vector [12]; anisotropy parameter ε [13]; and weighted $A_{\theta }$ parameter [14].

This paper introduces a novel method for studying the influence of crystalline anisotropy on the first magnetization curve and total magnetic energy loss. Specifically, the total magnetic energy loss curve is derived from the areas of the hysteresis loops, with the peak polarization values at given external magnetic fields defining the first magnetization curve. It is important to note that magnetic behavior varies between the rolling and transverse directions.

The proposed method includes the following: (i) saturation magnetization; (ii) external applied field vectors; and (iii) energies associated with these vectors within the vectorial space of the crystal unit cell.

This method is implemented through the following steps: (i) Electron Back-Scattered Diffraction (EBSD) analyses and Single-Sheet Tests (SSTs); (ii) EBSD data processing using an appropriate toolbox; (iii) energy considerations related to the magnetization process; and (iv) mathematical modeling of energy loss.

**Figure 1:**
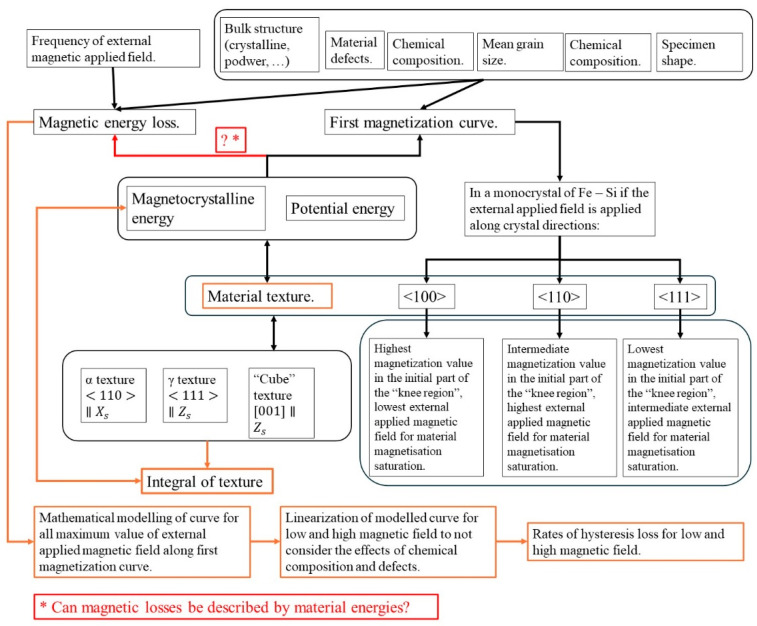
Conceptual map of the paper’s work.

Figure 1 presents a conceptual map of the work.

This approach could enable the development of more accurate models for predicting the magnetization curve and magnetic energy losses compared to existing methods. Furthermore, it will provide preliminary data to help establish guidelines for optimizing rolling processes and heat treatments in industrial plants.

## 2. Theoretical Bases

**Figure 2:**
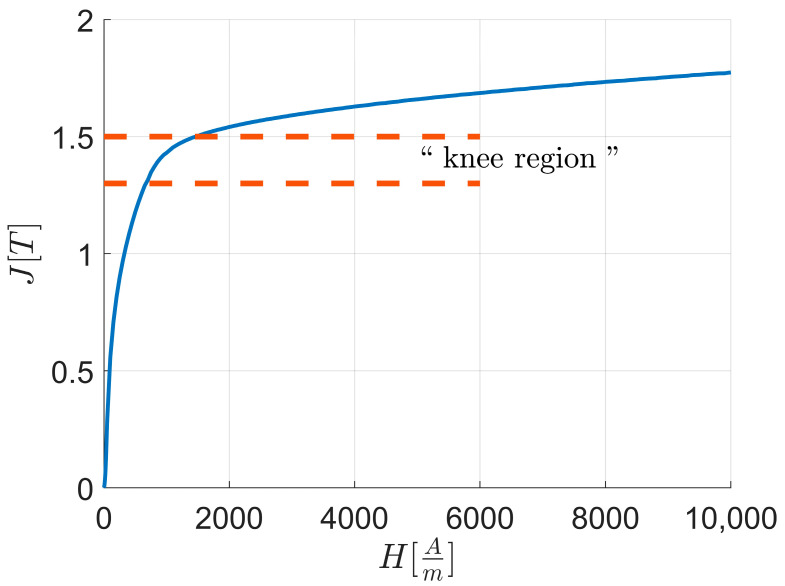
Magnetic polarization J vs. external applied magnetic field H and “knee region” highlighted.

In the initial part of the first magnetization curve up to the “knee region”, as shown in Figure 2, the magnetization process is primarily governed by the motion of domain walls. As the curve progresses through the “knee region”, the saturation magnetization vectors of the newly formed domains begin to rotate, aligning with the direction of the externally applied magnetic field. Eventually, at high external magnetic field values, the domain configuration reaches a state where all saturation magnetization vectors are parallel to the applied field direction.

For a single bcc crystal structure material subjected to an external magnetic field applied along one of the crystal directions <100>, <110>, and <111>, the magnetization value at the initial part of the “knee region” varies as follows: it is highest for <100>, lowest for <111>, and intermediate for <110>. However, the external magnetic field required to align all saturation magnetization vectors parallel to the applied field is lowest for <100>, highest for <110>, and intermediate for <111>.

The mean magnetization of a material is related to the variation in the Landau free energy. Specifically, under constant entropy and temperature conditions, it is given by the Helmholtz free energy, which reflects changes in the system’s internal energy. 

In an ideal bcc crystal, the potential energy of the saturation magnetization vector $E_{p}$, which drives the magnetization process up to the “knee region,” is given by:

(1) $$E_{p}=-\mu_{0}\;\;M_{s}\cdot H$$

where $M_{s}$ is the saturation magnetization vector; H is the external applied magnetic field vector; and $\mu_{0}$ is the vacuum permeability; the symbol denotes the scalar product between vectors.

However, the magnetization process up to saturation is also influenced by the magnetocrystalline anisotropy energy $E_{a}$.

(2) $$E_{a}=K_{0}+K_{1}\left(\alpha_{1}\alpha_{2}+\alpha_{1}\alpha_{3}+\alpha_{2}\alpha_{3}\right)+K_{2}\alpha_{1}^{2}\alpha_{2}^{2}\alpha_{3}^{2}$$

Here, $\alpha_{i}$ represents the direction cosine, which is the angle between the saturation magnetization vector and a specific crystal direction during its rotation toward the direction of the external applied field, where it reaches the minimum value of $E_{a}$. Specifically, the magnetocrystalline anisotropy energy $E_{a}$ follows the order $E_{a,<100>}<E_{a,<110>}<E_{a,<111>}$ when the saturation magnetization vectors are fully aligned with the external applied field [1,2,5,7,8].

Moreover, in materials like iron or Fe–Si alloys, the term $K_{2}$ could be neglected since $K_{1}\gg \left|{\;K}_{2}\right|$ and $K_{0}≅0$ [1,8,15].

The total energy of the ideal material during the magnetization process up to the “knee region” (*E*_*t*,1_), neglecting other energy contributions like the shape one, is as follows:

(3) $$E_{t,1}=E_{p}$$

While over the “knee region”, considering also the anisotropy magnetocrystalline energy, the total energy (*E*_*t*,2_) is calculated as follows:

(4) $$E_{t,2}=E_{a}+E_{p}$$

In real polycrystalline materials, the magnetization process is more complex due to factors such as the frequency of the external magnetic field and the specific crystal orientations of each grain. Additionally, materials often contain various defects—such as non-metallic inclusions, voids, and second phases—that act as pinning sites for domain wall motion. Despite these complexities, this study focuses on crystal orientations. Consequently, factors such as frequency, shape anisotropy, and inclusions are considered constant, as they are consistent across the specimens studied.

In polycrystals, lattice orientations can be described using Euler angles, which represent the rotation of the reference frame fixed to the unit crystal cell relative to the reference frame fixed to the specimen. The most comprehensive information about the distribution of crystal orientations in the material is provided by orientation distribution function (ODF) maps [15,16,17,18,19]. 

The ODFs for each orientation g are given by the orientation function distribution density $f\left(g\right)$.

(5) $$\frac{dV}{V}=f\left(g\right)dg$$

where dV is the volume of the crystal with orientation g; V is the total volume of the crystal; and dg is the orientation variation from g [15].

It is also possible to plot the ODF along a particular set of directions, obtaining a so-called fiber plot. 

To assess the relationships between energy contributions and texture analyses, two reference frames are considered: first, the sheet reference frame (RD, TD, and ND), as shown in Figure 7 (d2); second, the specimen reference frame $\left(X_{s},{\;Y_{s},\;Z}_{s}\right)$, as shown in Figure 7 (d3). The reference frame axes are aligned such that $RD∥X_{s}$, $ND∥Y_{s}$, and $TD∥Z_{s}$.

The most important rolled fibers for bcc crystal materials are α, with $<110>∥X_{s}∥RD$, and *γ*, with $<111>∥Z_{s}∥TD$. Specifically, the orientations are defined by a set of Euler angles that describe the rotation from the initial orientation $g_{i}$ to the final orientation $g_{f}$. For α and *γ* fibers the set of orientations are (Table 1):

In recrystallized Fe–Si alloys, the commonly considered texture components include the Goss component {110}<001> and the cube component {100}<001> [16,17,19,20]. However, this study will focus on analyzing the set of cubic orientations, also known as the “fiber Cube” (Table 2).

$Cube:\left[001\right]∥Z_{s}∥TD$, considering all the rotated cubes with respect to the $X_{s}∥RD$ and considering the magnetic behavior along the [100] and [010] axes. 

This assumption enables the calculation of $E_{t,2}$ along each principal crystal axis while varying only one Euler angle. Table 3 provides the anisotropy energy $E_{a}$ and potential energy $E_{p}$ along the rolling direction (RD):

In this context, $\beta_{1}$ is the angle between the external applied field H and the saturation magnetization $M_{s}$ along one of the easy axes (<100>), with $0\leq \beta_{1}\leq 45{}^{\circ}$, accounting for the symmetry of $M_{s}$ along the crystal axes; $\theta_{1}$ is the angle between $M_{s}$ and the <100> directions, with $0\leq \theta_{1}\leq 45{}^{\circ}$; $\theta_{2}$ is the angle between $M_{s}$ and the <100> directions, with $0\leq \theta_{2}\leq 45{}^{\circ}$; and $\delta_{1}$ is the misorientation angle between $M_{s}$ and H, given that H belongs to the {111} planes.

The definitions of the angles $\theta_{1}$ and $\theta_{2}$ imply the following relationships: $E_{a,Cube}<E_{a,\alpha }$; $E_{a,\alpha }=cost.=\max\left(E_{a}\right)$; and $E_{a,\gamma }=cost.=K_{0}$, $K_{0}≅0$. 

Thus, for the anisotropy energy: $E_{a,\gamma }<E_{a,Cube}<E_{a,\alpha }$.

Therefore, the α fiber is the one that generates the highest value of magnetocrystalline anisotropy energy opposing the alignment with the external magnetic field.

The potential energy is influenced by the $\beta_{1}$ angle. If $\beta_{1}<45{}^{\circ}$, then $E_{p,Cube\;}>E_{p,\alpha }$. 

Thus, it follows that: (i) the fewer the <100> directions that are misoriented with respect to the rolling direction (RD), the higher the potential energy of the magnetic moments and the lower the anisotropy field. As a result, the magnetization value at the beginning of the “knee region” is higher, and saturation magnetization can be achieved with a lower external magnetic field. (ii) The $E_{a}$ contribution of γ fiber can be neglected because $K_{0}≅0$ and only $E_{p}\neq 0$. (iii) $E_{a,\;\alpha \;}$ provides the highest energetic contribution. The higher its value, the lower the magnetization value at high external magnetic field intensities H.

Table 4 reports anisotropy $E_{a}$ and potential $E_{p}$ along TD. 

Where $\beta_{2}$ is the angle between H and $M_{s}$ along one of the easy axes, with $0\leq \beta_{2}\leq 45{}^{\circ}$ due to the symmetry of $M_{s}$ along the crystal axes; $\theta_{3}$ is the angle between $M_{s}$ and the <100> directions, with $0\leq \theta_{3}\leq \beta_{2}$; and $\delta_{2}$ is the misorientation angle between $M_{s}$ and H, where H is parallel to <111>. 

The definition of the angles $\theta_{3}$ implies that $E_{a,Cube}<E_{a,\alpha }<E_{a,\gamma }$. 

Thus, the γ fiber generates the highest magnetocrystalline anisotropy energy, opposing the alignment with the external magnetic field.

Regarding the potential energy: if $0\leq \beta_{2}<45{}^{\circ}$, then $E_{p,Cube}\geq E_{p,\alpha }$. 

Thus, the γ produces the highest magnetocrystalline anisotropy energy opposing the external applied field. This results in a reduced magnetization value at the beginning of the “knee region”, and the saturation magnetization can only be achieved at higher external magnetic field values compared to those along the rolling direction (RD). Additionally, the $E_{a}$ contribution of the “Cube” fiber can be neglected because ${\;K}_{0}≅0$ and $E_{p,\;Cube}=\max\left(E_{p}\right)$. 

Ultimately, because $E_{a,\;\gamma }$ provides the highest anisotropy contribution among the fibers, a higher value of $E_{a,\;\gamma }$ results in a lower magnetization value at high external magnetic field intensities H.

From these observations, anisotropy energy contributions can be assessed for an external magnetic field applied along specific crystal directions or within particular crystal planes. This leads to a simplification in studying the effects of anisotropy energies. 

To qualitatively assess the influence of textures on energy contributions, we consider a simplified analysis of the function $f\left(g\right)$ along a fiber. 

Given that $g\in R^{3},\;f:\;R^{3}\to R$, and since two angles remain constant while one angle varies along the fiber, we can define a function $h\left(\theta \right)$ where θ is the varying angle along the fiber, with θ∈R and h:R→R. 

The function $h\left(\theta \right)$ is not defined but is derived from $f\left(g\right)$ along a fiber.

To account for all fiber orientation contributions with one specific direction parallel to the specimen-fixed reference frame, a numerical integration of the function $h\left(\theta \right)-I$ is performed from the initial varying angle $\theta_{i}$ to the final angle $\theta_{f}$:

(6) $$I=\int_{\theta_{i}}^{\theta_{f}}h\left(\theta \right)\;\;d\theta \;$$

The study of the influence of crystallographic anisotropy on total energy loss can be conducted by describing the phenomenon using a mathematical function, following an identification process [21]. This function does not directly represent the physical processes of magnetism but provides a mathematical model useful for a preliminary analysis of how various factors affect the material’s behavior.

Given the shape of the total energy loss, a double exponential function is selected as the fitting model:

(7) $$y\left(x\right)=ae^{b\;x}+ce^{d\;x}$$

In this study, the rates of variation of total energy loss up to the “knee region” and in the rotating regions were chosen since the function could be linearized in two main regions.

The first region is around $H=0\;\left[\frac{A}{m}\right]$, with a McLaurin series until the first degree:

(8) $$y_{lin,1}\left(x\right)=\left(ab+cd\right)\;x+\left(a+c\right)$$

The coefficient of the linearized function, which represents the rate of variation in the first region, is:

(9) $$m_{lin,1}=(ab+cd)$$

The second region corresponds to the high value of H where the function can be approximated with a line with starting point $P_{1}=\left(H\equiv H_{ref};\;P_{s,fit}\left(H_{ref}\right)\right)$ and ending at point $P_{2}=\left(H\equiv H_{end}=10000\left[\frac{A}{m}\right];\;P_{s,fit}\left(H_{end}\right)\right)$: 

The linear approximating function is as follows:

(10) $$y_{lin,2}\left(x\right)=p_{1}x+p_{2}$$

The coefficient of the linearized function, which represents the rate of variation in the second region, is as follows:

(11) $$m_{lin,2}=p_{1}$$

## 3. Materials and Methods 

The materials studied were three rolled Fe–Si alloys of grade M350—50A, in accordance with EN 10106. Their chemical compositions were analyzed using an Optical Emission Spectrometer (OES ARL 346 (Thermo Fisher Scientific, 168 Third Avenue, Waltham, MA USA 02451)). The acronyms and compositions of the analyzed sheets are detailed in Table 5. The key alloying elements are C, Si, Mn, and Al, as they significantly impact the magnetization process, saturation polarization, energy loss, and magnetocrystalline anisotropy. Additionally, S and N influence the total energy loss [1,2,6,8,10].

Magnetic characterization was performed using a Brockhaus Messtechnik Single-Sheet Tester (SST) (BROCKHAUS MEASUREMENTS, Dr. Brockhaus Messtechnik GmbH & Co. KG, Lüdenscheid, Germany) model MPG100 D DC/AC. The device features an external magnetic field frequency range from 3 Hz to 10 kHz, with a maximum polarization of 2 T and measurement repeatability within ±2%.

The external magnetic field was applied along the rolling direction (RD) and transverse direction (TD) of the sheets at a frequency of 50 Hz.

Microstructural characterization was conducted with a scanning electron microscope (TESCAN MIRA3 FEG-SEM (TESCAN ORSAY HOLDING, Brno, Czech Republic)) equipped with energy-dispersive X-ray spectroscopy (EDS, Bruker Quantax 200/30 mm^2^ (Bruker Nano GmbH, Berlin, Germany)) and an electron backscattered diffraction (EBSD) detector (e–Flash HD (Bruker Nano GmbH, Berlin, Germany)). The crystallographic analyses were performed on the longitudinal cross-sections of the sheets and processed using the MATLAB R2024a Toolbox MTEX v5.10.0 [22]. Specifically, the EBSD data were pre-processed with the Half Quadratic filter to denoise and complete the data [23].

Before investigation, samples were embedded in conductive resin and polished according to standard metallographic procedures, achieving a finish with 50 nm colloidal silica.

## 4. Experimental Results

### 4.1. Magnetic Characterization

**Figure 3:**
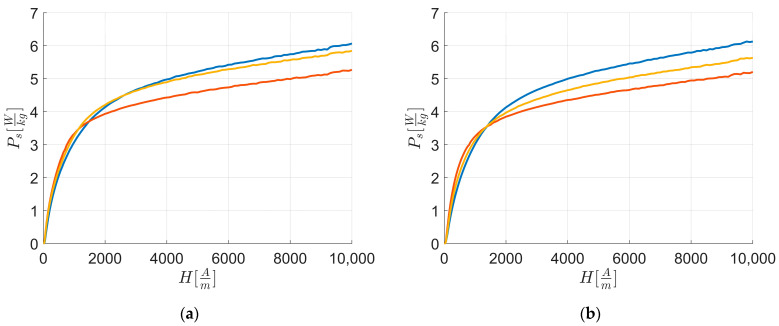
Magnetic characterization experimental energy loss $P_{s}$ vs. external applied magnetic field H: energy losses along RD (**a**) and TD (**b**). The colors are related to the behavior of: L1—blue; L2—orange; and L3—yellow.

Figure 3 illustrates the energy losses $P_{s}\left[\frac{W}{kg}\right]$ of all the materials in function of the intensity of the externally applied $H\left[\frac{A}{m}\right]$ field along RD and TD, as evaluated by the SST method. It highlights that at $H_{end}$: $P_{s}\left(L_{1}\right)>P_{s}\left(L_{3}\right)>P_{s}\left(L_{2}\right)$ in RD and TD.

The energy loss curves are derived from the value areas of hysteresis loops, whose magnetic polarization tips and corresponding external magnetic field intensities generate the first magnetization curve (Figure 5).

**Figure 4:**
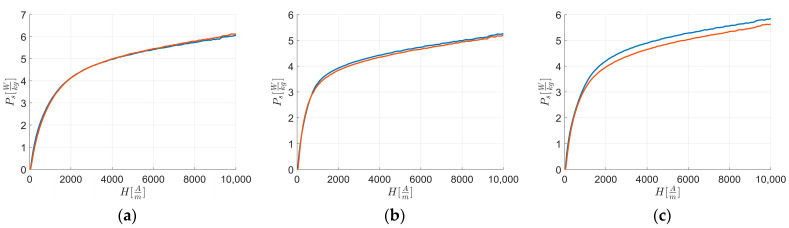
Magnetic characterization experimental energy loss $P_{s}$ vs. external applied magnetic field H: energy losses along RD (blue) and TD (orange). The figures are related to: (**a**) L1; (**b**) L2; and (**c**) L3.

Figure 4 presents the experimental energy losses for each material along the respective RD and TD. The curves show that $P_{s}$ is higher at $H_{end}$ only for L1 along TD than RD.

Table 6 summarizes the differences in magnetic energy loss along RD and TD at $H_{end}$. L1 exhibits a negative difference, indicating higher energy loss along TD compared to RD, while L3 has the highest difference between RD and TD. 

**Figure 5:**
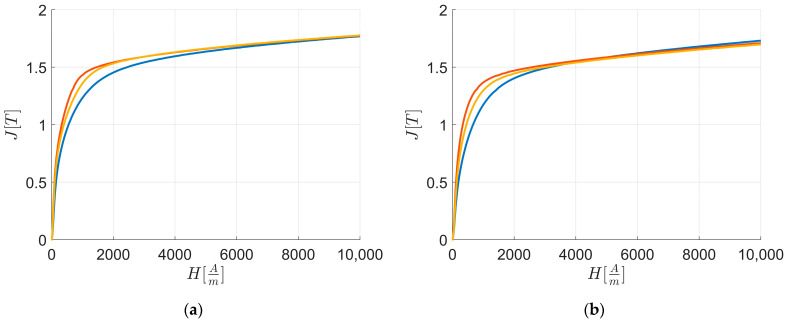
Magnetic characterization polarization J vs. external applied magnetic field H: first magnetization curves along RD (**a**) and TD (**b**). The colors are related to the behavior of: L1—blue; L2—orange; and L3—yellow.

Figure 5 displays the experimental first magnetization curves along RD and TD up to $H_{end}$.

Table 7 provides the polarization J[T] at $H_{end}$. The data show that $J\left(L_{3}\right)>J\left(L_{2}\right)>J\left(L_{1}\right)$ along RD while $J\left(L_{1}\right)>J\left(L_{2}\right)>J\left(L_{3}\right)$ along TD.

**Figure 6:**
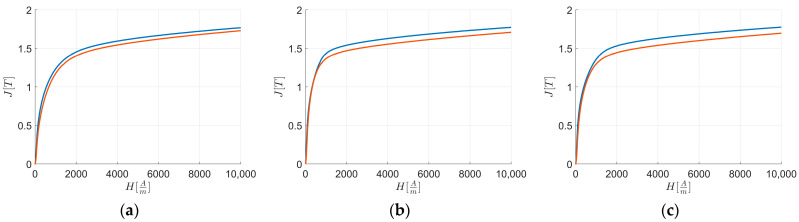
Magnetic characterization polarization J vs. external applied magnetic field H: first magnetization curves of L1 (**a**); L2 (**b**); and L3 (**c**) along RD (blue) and TD (orange).

Figure 6 presents the experimental first magnetization curves for each material along RD and TD. The graphs show that for all the sheets J[T] at $H_{end}$ is higher along RD compared to TD. 

Table 8 reports the differences in polarization ΔJ along RD and TD at $H=H_{k}=700\left[\frac{A}{m}\right]$, considered the initial point of the “knee region”. It shows that polarization along RD is consistently higher than along TD at $H_{k}$.

Table 9 presents the differences in polarization between all sheets along RD and TD at $H_{k}$. The results indicate that L2 shows the greatest differences in magnetization compared to the other sheets.

### 4.2. Crystallographic Characterisations

**Figure 7:**
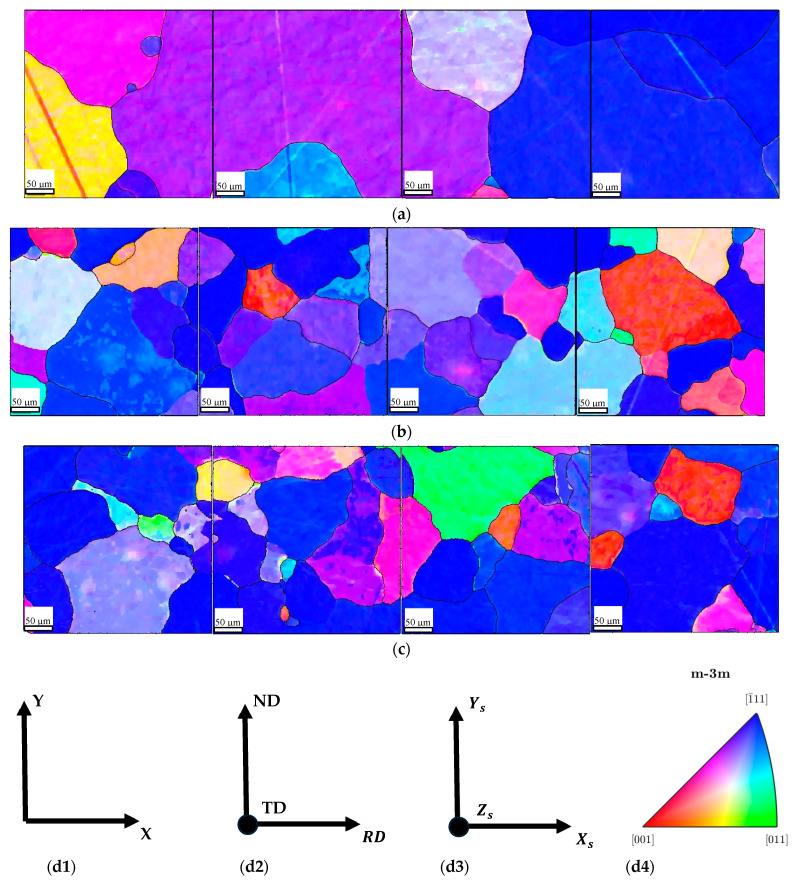
Orientations reconstruction of sheets, micrometric notch of 25 μm: (**a**) L1; (**b**) L2; (**c**) L3; (**d1**) the coordinate system of the orientation map; (**d2**) the sheet reference frame; (**d3**) the fixed specimen reference frame for the orientation analyses; and (**d4**) the IPF colorkey map.

Figure 7 summarizes the results of EBSD analyses, processed with the MATLAB Toolbox MTEX. It shows that the [111] direction (blue) aligned with the normal direction of the transverse section is predominant in all sheets. Additionally, some grains with [001] (red) and [011] (green) directions are present in L2 and L3. 

## 5. Data Analyses

Table 10 lists the integral values I of each fiber for each sheet. The data show that L2 has the highest $I_{Cube}$, L3 the highest $I_{\gamma }$, and L1 has the highest $I_{\alpha }$.

The fitting parameters for Function (7) were obtained (Table 11) by solving a nonlinear least-squares problem [21,24] using various types of functions and employing a Trust Region Algorithm [24] via the MATLAB R2024a Curve Fitting Toolbox.

The goodness of fit for the experimental data was evaluated using the sum of squared errors (SSE) [24] and the root mean squared errors (RMSE) [25] to determine if the Trust Region Algorithm successfully converged to the optimal minimum. Additionally, the coefficient of determination ${\bar{R}}^{2}$ was calculated to assess the correlation between the experimental data and the fitting curve [26], with particular emphasis on the adjusted ${\bar{R}}^{2}$. This adjusted ${\bar{R}}^{2}$ accounts for the number of fitting parameters and helps identify potential overfitting [27].

The low values of SSE and RMSE, along with very high values of ${\bar{R}}^{2}$ (Table 12), confirm a good fit between the experimental and calculated data. Thus, Function (7) accurately describes the magnetic energy loss behavior of the material along the rolling direction (RD) and transverse direction (TD) and is suitable for studying the material’s magnetic anisotropy as a function of the intensity of the applied external field. 

**Figure 8:**
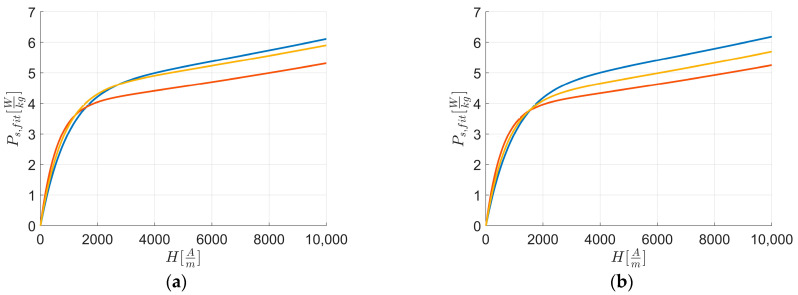
Fitted curves of experimental data of magnetic energy loss $P_{s,fit}$ vs. external applied magnetic field H along RD (**a**) and TD (**b**). The colors are related to the behavior of: L1—blue; L2—orange; and L3—yellow.

**Figure 9:**
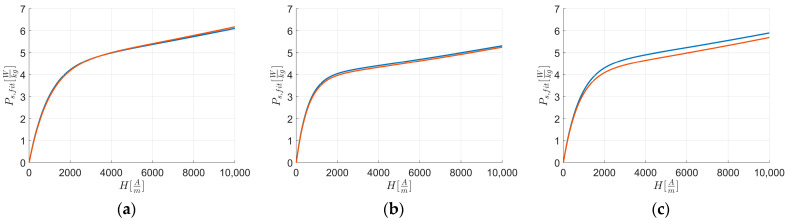
Fitted curves of experimental data of magnetic energy loss $P_{s,fit}$ vs. external applied magnetic field H along RD (blue) and TD (orange). The figures are related to: (**a**) L1; (**b**) L2; and (**c**) L3.

Figure 8 reports the fitted curves $P_{s,\;fit}$ of Figure 3, while Figure 9 reports the fitted curves of Figure 4. 

Based on the above, Function (7) can be reformulated as follows:

(12) $$P_{s,fit}\left(H\right)=ae^{b\;H}+ce^{d\;H}$$

The linear coefficient values of Function (8) are reported in Table 13 for all the energy loss fitted curves, indicating that $m_{RD}>m_{TD}$ for all the sheets.

Table 14 provides the SSE, RMSE, and ${\bar{R}}^{2}$ values for $H\geq H_{ref}$, where $H_{ref}$ is the field strength at which the experimental curves become linear. The SSE and RMSE values indicate that the linear function (10) accurately fits the data provided by Function (7) for $H\geq H_{ref}$, while the ${\bar{R}}^{2}$ values confirm a strong correlation between the experimental and fitted data.

The procedure for finding the best line approximation is the same as applied to Function (7). The values of linear coefficients for the fitting linear Function (10) are reported in Table 15, showing that $m_{RD}<m_{TD}$ for all the sheets.

## 6. Discussion 

Based on the results of the first magnetization curves along the rolling direction (RD) and transverse direction (TD) (Figure 5 and Figure 6), and considering Equations (1) and (3) along with Table 3, Table 4, Table 8, Table 9 and Table 10, the following conclusions can be drawn for $H\leq H_{k}$ where domain wall motion is the predominant mechanism:

(13) $$E_{p}=E_{p,\alpha }+E_{p,\gamma }+E_{p,Cube}$$

where

$$E_{p,Cube}=-M_{s}H;E_{p,\alpha }=-M_{s}Hcos\left(45{}^{\circ}\right);E_{p,\gamma }=-M_{s}Hcos\left(54.7{}^{\circ}\right).$$

Thus,

$$E_{p,Cube}>E_{p,\alpha }>E_{p,\gamma }$$

and

$$J_{Cube}\left(H_{k}\right)>J_{\alpha }\left(H_{k}\right)>J_{\gamma }\left(H_{k}\right).$$

At $H_{k}$, considering the integral of textures along RD and TD:

$$I_{\gamma }+I_{\alpha }+I_{Cube}\left(L_{1}\right)<I_{\gamma }+I_{\alpha }+I_{Cube}\left(L_{2}\right)<I_{\gamma }+I_{\alpha }+I_{Cube}\left(L_{3}\right).$$

Hence,

$$E_{p}\left(L_{1}\right)<E_{p}\left(L_{2}\right)<E_{p}\left(L_{3}\right).$$

However,

$$E_{p,Cube}>E_{p,\alpha }>E_{p,\gamma },I_{Cube}\left(L_{2}\right)>I_{Cube}\left(L_{3}\right)>I_{Cube}\left(L_{1}\right).$$

Thus,

$$J_{L_{2}}\left(H_{k}\right)>J_{L_{3}}\left(H_{k}\right)>J_{L_{3}}\left(H_{k}\right)$$

Despite $I_{\gamma }+I_{\alpha }\left(L_{3}\right)>I_{\gamma }+I_{\alpha }\left(L_{1}\right)$, the significant difference $I_{Cube}\left(L_{3}\right)\gg I_{Cube}\left(L_{1}\right)$ explains the results. 

These observations are supported by polarization differences along RD and TD for $H=H_{k}$. 

Figure 5 points out that the first magnetization curve of L2 consistently shows higher polarization values than L1 and L3, with L3 being higher than L1, and L1 having the lowest polarization values among the curves. Table 9 shows these differences for $H=H_{k}$, and the texture integral values from Table 10 confirm the results from the previous equations. 

Figure 6 highlights that for $H=H_{k}$ the polarization values are consistently lower along TD than RD. Table 8 illustrates these differences for $H=H_{k}$, and the texture integral values from Table 10 further confirm the results from the previous equations.

For intensities of external applied field lower than $H_{k}$ for each sheet, denoted as $H_{low}$, the following expression is used:

$$J\propto E_{t}\to \Delta J\propto \Delta E_{t}.$$

If

$$E_{p,RD}\left(H_{low}\right)=E_{p,TD}\left(H_{low}\right)\mathrm{and}\;\Delta J>0$$

Then,

$$E_{a,RD}\left(H_{low}\right)<E_{a,TD}\left(H_{low}\right)$$

Thus,

$$J_{RD}\left(H_{low}\right)>J_{TD}\left(H_{low}\right).$$

These observations are supported by polarization differences along RD and TD for $H<H_{k}$. 

From Figure 6, it is evident that from very low values of external applied field intensity, the polarization values are consistently lower along TD than RD. The texture integral values from Table 10 verify the results from the previous equations.

These observations demonstrate that higher Cube texture integral values correspond to higher magnetization at the onset of the “knee region”, indicating that less energy is required for magnetization with this texture and that magnetocrystalline anisotropy also plays a role at relatively low magnetic fields.

For $H\to H_{end}$, considering Equation (4), Table 3, Table 4, Table 7 and Table 10, along with Figure 5 and Figure 6, where $E_{p}$ is approximately constant for all sheets, the following expression is used:

(14) $$E_{a}=E_{a,\alpha }+E_{a,\gamma }+E_{a,Cube}$$

Along RD: 

The following is given:

$$E_{a,\gamma }≅0,\;\mathrm{then}\;E_{a,RD}=E_{a,\alpha }+E_{a,Cube}.$$

Since

$$I_{\alpha }+I_{Cube}\left(L_{3}\right)<I_{\alpha }+I_{Cube}\left(L_{2}\right)<I_{\alpha }+I_{Cube}\left(L_{1}\right).$$

Thus,

$$E_{a,RD}\left(L_{1}\right)>E_{a,RD}\left(L_{2}\right)>E_{a,RD}\left(L_{3}\right).$$

Therefore,

$$J_{L_{3}}\left(H_{end}\right)>J_{L_{2}}\left(H_{end}\right)>J_{L_{1}}\left(H_{end}\right).$$

These results are confirmed by polarization values at maximum external fields along RD. Figure 5a highlights that for $H\to H_{end}$, the polarization values are the highest for L3, lower for L2, and the lowest for L1. Table 7 shows these differences for $H\to H_{end}$, and the texture integral values from Table 10 prove the results from the previous equations, confirming that lower Cube and α texture integral values correspond to higher magnetization at maximum magnetic field intensity.

Along TD: 

Given that $E_{a,Cube}≅0$,

$$E_{a,TD}=E_{a,\alpha }+E_{a,\gamma }.$$

Since

$$I_{\alpha }+I_{\gamma }\left(L_{1}\right)<I_{\alpha }+I_{\gamma }\left(L_{2}\right)<I_{\alpha }+I_{\gamma }\left(L_{3}\right).$$

Thus,

$$E_{a,TD}\left(L_{3}\right)>E_{a,TD}\left(L_{2}\right)>E_{a,TD}\left(L_{1}\right).$$

Therefore,

$$J_{L_{1}}\left(H_{end}\right)>J_{L_{2}}\left(H_{end}\right)>J_{L_{3}}\left(H_{end}\right).$$

These results are confirmed by polarization values at maximum external fields along TD. Figure 5b points out that for $H\to H_{end}$ along TD, the polarization values are the highest for L1, lower for L2, and the lowest for L3. Table 7 shows these differences for $H\to H_{end}$, and the texture integral values from Table 10 support the results from the previous equations, confirming that lower Cube and γ texture integral values correspond to higher magnetization at the maximum field intensity. 

Hence, anisotropy energy is lower at high magnetic field intensities, and the material exhibits less isotropic behavior.

According to [1,5,7], increasing silicon content in Fe–Si alloys generally increases electrical resistivity and should decrease energy loss. However, experimental data show the highest energy loss for L1 (1.879% Si), the lowest for L2 (1.718% Si), and intermediate for L3 (1.971% Si) at $H_{end}$ along RD and TD (Figure 1). This discrepancy suggests that the magnetic behavior of Fe–Si alloys should also consider the effects of N, S, Al, and Mn (Table 5). Specifically, elements like AlN can increase magnetic energy loss. Given the low and nearly constant S content, the presence of AlN is significant: L1, with the highest Al and N content (0.403% Al, 0.0086% N), exhibits the worst magnetic behavior; L3, with low Al and intermediate N (0.353% Al, 0.0042% N), shows intermediate behavior; and L2, with intermediate Al and the lowest N (0.397% Al, 0.003% N), demonstrates the best magnetic behavior.

Examining the initial part of the loss curve (Figure 8 and Figure 9) and using Equation (1), Equation (3), and Equation (8), the following can be derived: $P_{s,\;fit}\;\propto J\left(H\right)$.

Therefore,

$$\Delta P_{s,fit}\propto \Delta J\left(H\right)\;\mathrm{and}\;\underset{H\to 0}{\lim}\frac{\Delta J\left(H\right)}{\Delta H}≅\mu_{i}^{\prime }=cost;$$

The following is given:

$$\underset{H\to 0}{\lim}\frac{\Delta P_{s,fit}\left(H\right)}{\Delta H}=m_{lin,1}=cost.$$

Thus, 

$\mu_{i}^{\prime }\;\propto \;m_{lin,1}$, with $\mu_{i}^{\prime }$ being the initial differential permeability.

Assuming homogeneous defect distribution and nearly constant electrical resistivity, at H→0, the following can be derived:

$$\mu_{L1}^{\prime }\neq \mu_{L2}^{\prime }\neq \mu_{L3}^{\prime }\;\mathrm{and}\;E_{p}=cost.$$

Thus,

$$E_{a}\neq 0.$$

From Figure 8, it is evident that along RD and TD for $H\leq H_{k}$ the magnetic loss curve of L2 consistently shows higher magnetic loss values than L1 and L3, with L3 being higher than L1, and L1 having the lowest magnetic loss values among the curves. By linearizing the first magnetization curves (Figure 4 and Figure 5) with an equation similar to Equation (8) from H→0 up to $H_{k}$, to avoid instrumental acquisition errors at very low fields, it is observed that higher $J\left(H_{k}\right)$ correlates with a higher loss rate, as indicated by Equation (9) and supported by Table 13. Based on linear coefficients (Table 15) and the previous observations of the first magnetization curve and loss rate for $H<H_{k}$, anisotropy energy significantly influences magnetic energy loss behavior. 

From Figure 9, it is evident that for $H\geq H_{ref}$ the magnetic loss curve of L1 consistently shows higher magnetic loss values along TD than RD compared to other sheets, while the others always have lower values along TD than RD. Table 15 shows these differences for rates of loss for $H\geq H_{ref}$, and together with Table 6 and the texture integral values from Table 10, it is confirmed that texture energies impact L1 more than other sheets for $H\geq H_{ref}$ along RD and TD.

The results presented represent a novel method for describing magnetic behavior in the vectorial space of the crystal unit cell, involving saturation magnetization, external applied field vectors, and associated energies for typical fiber texture orientations in rolled materials. This approach offers a new perspective compared to semi-empirical methods. Further research is needed to explore the contributions of different orientations to the magnetization and energy loss of materials.

## 7. Conclusions

The aim of this paper is to evaluate the effect of material texture on the magnetization and magnetic energy loss of Fe–Si alloys by considering the definitions of potential and magnetocrystalline anisotropy magnetization energy in magnetic materials. This study integrates the orientation distribution function along each fiber and models energy loss as a function of the externally applied field.

The results highlight the following key points:

1. Impact of texture on energy loss: the texture of the material significantly influences the first magnetization curve and the initial energy loss. According to Table 13, the rate of variation in energy loss between the rolling direction (RD) and the transverse direction (TD) is 2.04% for each specimen. Specifically, along the RD, the energy loss rates are as follows: 46.93% between L1 and L2; 15.51% between L1 and L3; and 19.44% between L2 and L3. Along the TD, the rates are: 32.4% between L1 and L2; 15.79% between L1 and L3; and 19.72% between L2 and L3.
2. Variation in energy loss with polarization: although the maximum polarizations achieved by L3 and L2 are similar along RD and TD, their energy loss behavior differs significantly with the applied external field. Table 7 shows that the polarization difference between L2 and L3 is 0.17% along RD and 0.67% along TD. The variation in energy loss rates between L2 and L3 is 9.09% along RD and 12.64% along TD. Additionally, the polarization difference between RD and TD for the same specimen is 0.0648 T for L2 and 0.0795 T for L3 (Table 7). The corresponding variation in energy losses between RD and TD is 0.0639 W/kg for L2 and 0.2057 W/kg for L3 (Table 6). This results in a 1.38% variation in energy loss relative to the maximum polarization for L2 and a 61.35% variation for L3, indicating that the first magnetization curve does not directly influence the energy loss behavior.
3. Modeling energy loss with double exponential curve: The influence of texture on energy loss can be effectively studied using a double exponential curve, which provides insight into the contributions of different textures.
4. Vectorial space analysis: describing saturation magnetization, external applied field vectors, and the related energies in the vectorial space of the crystal unit cell—based on a typical fiber texture set of orientations for the rolled material—proves useful for analyzing both magnetization and magnetic energy losses.
5. These findings underscore the complex interplay between material texture and magnetic behavior, offering a detailed understanding of how different textures affect magnetization and energy loss in Fe–Si alloys.

## Figures and Tables

**Table 1:** Set of Euler angles of fibers α and γ.

Fiber	$g_{i}-g_{f}$
α	$\left[0{}^{\circ},0{}^{\circ},45{}^{\circ}\right]-\left[0{}^{\circ},90{}^{\circ},45{}^{\circ}\right]$
γ	$\left[60{}^{\circ},54.7{}^{\circ},45{}^{\circ}\right]-\left[90{}^{\circ},54.7{}^{\circ},45{}^{\circ}\right]$

**Table 2:** Set of Euler angles of fiber Cube.

Fiber	$g_{i}-g_{f}$
*Cube*	$\left[0{}^{\circ},0{}^{\circ},0{}^{\circ}\right]-\left[90{}^{\circ},0{}^{\circ},0{}^{\circ}\right]$

**Table 3:** Anisotropy (*E_a_*) and potential energies (*E_p_*) along RD.

	$E_{a}$	$E_{p}$
Cube	$K_{0}+K_{1}\sin^{2}\left(2\;\theta_{1}\right)$	$-M_{s}\;H\cos\left(\beta_{1}-\theta_{1}\right)$
α	$K_{0}+K_{1}\sin^{2}\left(2\;\theta_{2}\right)$	$-M_{s}\;H\cos\left(45{}^{\circ}-\theta_{2}\right)$
γ	$K_{0}$	$-M_{s}\;H\cos\left(\delta_{1}\right)$

**Table 4:** Anisotropy (*E_a_*) and potential (*E_p_*) energies along TD.

	$E_{a}$	$E_{p}$
Cube	$K_{0}$	$-M_{s}\;H$
α	$K_{0}+K_{1}\sin^{2}\left(2\;\theta_{3}\right)$	$-M_{s}\;H\cos\left(\beta_{2}-\theta_{3}\right)$
γ	$K_{0}+K_{1}\left(\alpha_{1}\alpha_{2}+\alpha_{1}\alpha_{3}+\alpha_{2}\alpha_{3}\right)+K_{2}\alpha_{1}^{2}\alpha_{2}^{2}\alpha_{3}^{2}$	$-M_{s}\;H\cos\left(\delta_{2}\right)$

**Table 5:** Chemical composition (weight %) of the studied Fe—Si alloys.

Sample Acronym	% C	% Si	% Mn	% Al	% S	% N	% Other	% Fe
L1	0.0036	1.879	0.245	0.403	0.004	0.0086	0.0383	Bal.
L2	0.0062	1.718	0.270	0.397	0.005	0.003	0.1031	Bal.
L3	0.0041	1.971	0.290	0.353	0.004	0.0042	0.0581	Bal.

**Table 6:** Total energy loss differences between RD and TD for all the sheets.

	$\Delta P_{s,fit}(H_{end})$
L1	−0.0728
L2	0.0639
L3	0.2057

**Table 7:** Polarization values at the maximum value of the externally applied field along RD and TD.

	$J\left[T\right]$	
	RD	TD
L1	1.7684	1.7302
L2	1.7740	1.7092
L3	1.7771	1.6976

**Table 8:** Differences of polarizations between RD and TD for each sheet.

	$\Delta J\left(H_{k}\right)$
L1	0.0595
L2	0.0637
L3	0.0617

**Table 9:** Differences of polarization along RD and TD between all the sheets.

		$\Delta J\left(H_{k}\right)$
L1–L2	RD	−0.2192
	TD	−0.2578
L1–L3	RD	−0.1242
	TD	−0.1545
L2–L3	RD	0.0950
	TD	0.1033

**Table 10:** Values of fiber integrals.

	$I_{\alpha }\cdot 10^{3}$	$I_{\gamma }\cdot 10^{3}$	$I_{Cube}$
L1	1.53	2.15	0.0041
L2	0.88	2.92	174.34
L3	0.72	4.4	27.87

**Table 11:** Values of fitting parameters of Function (7).

		a	$b\cdot 10^{-5}$	c	d
L1	RD	4.4580	3.1488	−4.4923	−0.0011
	TD	4.4537	3.2769	−4.4916	−0.001
L2	RD	3.9032	3.0893	−3.9533	−0.0018
	TD	3.8210	3.1811	−3.8840	−0.0018
L3	RD	4.3769	2.9879	−4.4113	−0.0013
	TD	4.0893	3.3129	−4.1344	−0.0013

**Table 12:** Values of goodness of fitting and correlation parameters of Function (7).

		SSE	RMSE	${\bar{R}}^{2}$
L1	RD	0.5406	0.066	0.9990
	TD	0.2903	0.0484	0.9995
L2	RD	0.3934	0.0563	0.9990
	TD	0.6769	0.0739	0.9982
L3	RD	0.5388	0.0659	0.9989
	TD	0.5805	0.0684	0.9987

**Table 13:** Linear coefficient of Function (8).

	$m_{lin,1}$	
	RD	TD
L1	0.0049	0.0048
L2	0.0072	0.0071
L3	0.0058	0.0057

**Table 14:** Values of SSE, RMSE, and ${\bar{R}}^{2}$ for each sheet for fitted magnetic energy loss with Function (5).

		SSE⋅10^−4^	RMSE	${\bar{R}}^{2}$
L1	RD	1.22	0.0078	0.9997
	TD	1.78	0.0094	0.9996
L2	RD	2.92	0.0121	0.9994
	TD	3.2	0.0127	0.9990
L3	RD	0.54	0.0061	0.9998
	TD	2.05	0.01	0.9995

**Table 15:** Values of Equation (11).

	$m_{lin,2}$	
	$\mathrm{RD}\cdot 10^{-4}$	$\mathrm{TD}\cdot 10^{-4}$
L1	1.85	1.95
L2	1.5	1.52
L3	1.65	1.74

## Data Availability

The raw data supporting the conclusions of this article will be made available by the authors on request.
